# A Retinal Vessel Segmentation Method Based on the Sharpness-Aware Minimization Model

**DOI:** 10.3390/s24134267

**Published:** 2024-06-30

**Authors:** Iqra Mariam, Xiaorong Xue, Kaleb Gadson

**Affiliations:** School of Electronics and Information Engineering, Liaoning University of Technology, Jinzhou 121001, China; mariamsaleem466@gmail.com (I.M.); japhgad@gmail.com (K.G.)

**Keywords:** retinal vessel segmentation, sharpness-aware minimization (SAM), RF-UNet, DRIVE dataset, medical image segmentation

## Abstract

Retinal vessel segmentation is crucial for diagnosing and monitoring various eye diseases such as diabetic retinopathy, glaucoma, and hypertension. In this study, we examine how sharpness-aware minimization (SAM) can improve RF-UNet’s generalization performance. RF-UNet is a novel model for retinal vessel segmentation. We focused our experiments on the digital retinal images for vessel extraction (DRIVE) dataset, which is a benchmark for retinal vessel segmentation, and our test results show that adding SAM to the training procedure leads to notable improvements. Compared to the non-SAM model (training loss of 0.45709 and validation loss of 0.40266), the SAM-trained RF-UNet model achieved a significant reduction in both training loss (0.094225) and validation loss (0.08053). Furthermore, compared to the non-SAM model (training accuracy of 0.90169 and validation accuracy of 0.93999), the SAM-trained model demonstrated higher training accuracy (0.96225) and validation accuracy (0.96821). Additionally, the model performed better in terms of sensitivity, specificity, AUC, and F1 score, indicating improved generalization to unseen data. Our results corroborate the notion that SAM facilitates the learning of flatter minima, thereby improving generalization, and are consistent with other research highlighting the advantages of advanced optimization methods. With wider implications for other medical imaging tasks, these results imply that SAM can successfully reduce overfitting and enhance the robustness of retinal vessel segmentation models. Prospective research avenues encompass verifying the model on vaster and more diverse datasets and investigating its practical implementation in real-world clinical situations.

## 1. Introduction

Color fundus images are easy to obtain, and retinal blood vessels can be observed without the need for any radiographic equipment [1]. The retinal vasculature is a biological feature that carries rich biological information and therefore plays an important role in many fields [2]. Due to the uniqueness of the retinal blood vessels of different individuals, they can be used as identification to build an individual identification system [3]. The retina is an extension of the cerebrum that shares its embryonic pathways and blood vessels [4]. Ophthalmologists use retinal vasculature to diagnose diseases involving damage to blood vessels and the vascular system, thereby explaining diabetic retinopathy (DR) [5] and diabetic macular disease (MD) [6]. Vascular information is considered important for developing computer-assisted automated systems to analyze DR and search for potential biomarkers of diabetes-related eye disease [7]. Therefore, vessel segmentation is an important step before the geometric properties of retinal vessels can be assessed [8]. A variety of machine learning software tools and methods have been developed for quantitative assessment of retinal vasculature [9,10,11,12,13]. However, widespread use of these tools has been limited due to the need for manual entry (IVAN [14], SIVA [15], and VAMPIRE [16]), their time-consuming nature (IVAN [14] and SIVA [15]), their applicability to only specific retinal regions (IVAN [14] and SIVA [15]), or their limited number of measurement parameters (IVAN [14], VAMPIRE [16], and QUARTZ [17,18]).

From previous years, different approaches have been proposed for blood vessel detection. They are mainly divided into two types: manual segmentation and algorithmic segmentation. The manual way is time-consuming and requires highly qualified technical personnel. Therefore, automated segmentation of retinal vessels, which is highly advanced and more accurate than manual segmentation, is highly demanded [19]. Automated algorithmic segmentation can be divided into two categories based on whether deep learning is used or not. The first category adopts traditional image processing techniques and machine learning methods like vascular tracking [20], morphological processing [21], and so on. These algorithms depend on earlier information and manually designed vessel segmentation features. Even though good results can be attained, these methods [22] have inadequate feature representation capabilities and versatility, making it hard to oversee complex vascular changes and structures.

Deep learning techniques, especially convolutional neural networks (CNNs), have shown promising results for segmenting retinal circuits. Models such as U-Net [23], RF-UNet [24], and their variants have been widely used for this task. These models learn to extract relevant features from retinal images and classify pixels as vascular or nonvascular based on these features. Some early vessel segmentation methods based on deep learning divided an image into patches and predicted all class labels of the patches’ pixels using a network composed of convolutional layers and fully connected layers [25]. But later, Long et al. [26] showed the state-of-the-art results with fully convolutional networks trained end-to-end and pixel-to-pixel. After the proposition of U-Net for image segmentation, different researchers took advantage of its architecture and created different variants specific to vessel segmentation. For example [3] used a U-Net structure and added an attention model to capture global information and enhance features. Also, ref. [27] integrated U-Net with patch-based loss weight mapping for retinal blood vessel segmentation to alleviate the problem of inconsideration of the background pixels in fundus images.

Currently, there have been several methods that have been proposed that outperform state-of-the-art approaches on various datasets. For example, to overcome the problem of convolutional neural networks failing to fully capture the global structure of retinal vessels and maintaining segmentation continuity, ref. [28] proposed a network that combines graph convolutional networks (GCNs) and attention mechanisms. As a result, the model’s segmentation accuracy was greatly increased because the model considered pixel relationships and learned vessel graphical structures. Ref. [29] proposed a hierarchical full-resolution fusion network (HFRF-Net) to accurately segment retinal blood vessels by preserving the spatial details of low-level blood vessels that are always lost due to the downsampling used by CNNs in order to obtain high-level contextual semantics. This method achieved high segmentation performance compared to other state-of-the-art methods. Also, to address the problem of the loss of crucial information and less segmentation accuracy due to the management of inadequate processing of local context features caused by skip connections in U-Net models, ref. [30] proposed a novel method called a multi-scale attention fusion network (MsAF-Net) for retinal vessel segmentation that adds two blocks—namely, an MsFE block and an MsAF block—between the encoder and decoder at each layer of the U-Net backbone. The MsFE block collects low-level features at different scales, and the output of the MsAF block replaces the skip connection in the U-Net backbone. Lastly, to enhance feature extraction, Kong et al. [31] proposed a method called RVS-FDSC that uses four-directional strip convolution instead of square convolution kernels for feature extraction.

Despite these advancements, there is still work to be done to improve generalization of the models. Due to the sharpness of training loss in most retinal vessel segmentation models, generalization is not optimal. Therefore, there is a need to introduce a method to tackle this issue.

According to previous studies, it has been seen that the sharpness of the training loss (how fast it changes in some neighborhoods around the parameters of the model) corresponds to the generalization error [32]. Therefore, minimizing the sharpness can significantly improve generalization capability. Sharpness-aware minimization (SAM) is a recent training method [33] that relies on worst-case weight perturbations and significantly improves generalization in various settings. It makes the surface of the loss function smoother and more generalized. Hence, there is no need to deal with min–max objectives like with adversarial learning [34]. Instead, it leverages linear approximation for better efficiency. Since its introduction, SAM has been widely used in the field of computer vision to improve the generalization of models. For example, refs. [35,36,37] all conducted their experiments on SAM for classification of images, and their results showed there was improvement in accuracy when SAM was added. Ref. [38] applied SAM to a backdoor defense model to enhance fine tuning. Zhou et al. [39] applied SAM on a deep long tailored recognition model, and the model achieved competitive performance compared to the state-of-the-art, and Wei et al. [40] used SAM to improve adversarial robustness.

In this paper, we propose the use of sharpness-aware minimization (SAM) as an alternative strategy for retinal vessel segmentation because traditional optimization techniques such as stochastic gradient descent (SGD) and Adam update model parameters based solely on the gradient of the loss function. However, these methods may struggle to navigate complex loss landscapes, potentially leading to suboptimal solutions and poor generalization performance. The main contributions of our paper are as follows:

- We propose an optimization algorithm that uses sharpness-aware minimization in the domain of medical image segmentation (MIS), specifically focusing on retinal-vessel segmentation.
- Comprehensive experiments are performed on the DRIVE dataset. The experimental results demonstrate that our proposed method produces better results on the validation data than the training data, which means our model generalizes better and, hence, has no overfitting.
- We integrate SAM into the RF-UNet architecture, which is a novel deep learning model for vessel segmentation, and the results are improved generalization of RF-UNet.

The rest of our paper is organized as follows: Section 2 explains the derivation of the SAM procedure and shows the full algorithm in detail. Experiments and analyses based on the DRIVE dataset are in Section 3. Finally, Section 4 provides the conclusion.

## 2. Methodology

In this section, we outline our approach, which is based on [41]’s work. We adopt their methodology to fit the integration of sharpness-aware minimization into the RF-UNet architecture. We use the representations b, b,B,B, and := to denote scalars, vectors, matrices, sets, and equality by definition, respectively.

**Figure 1:**
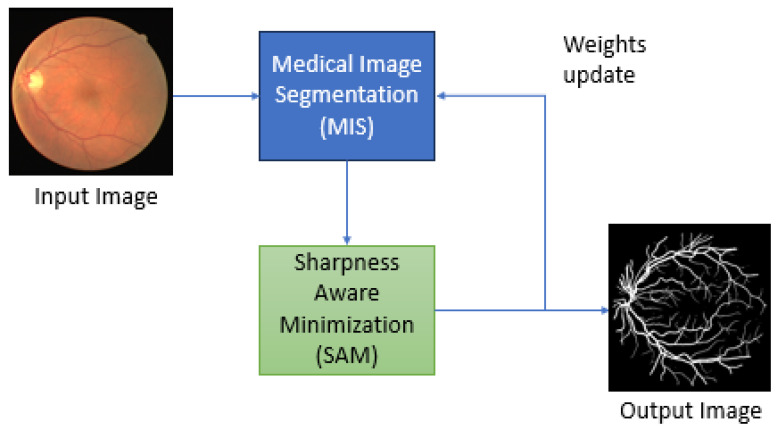
Block diagram showing how to integrate SAM into an MIS model.

During training, the FR-UNet parameters are initialized with a vector $w\;\epsilon \;W\subseteq R^{d}$ and the loss function $l:W\times X\times Y\to R_{+}$. Then, a training batch $S:=\cup_{i=1}^{n}\left(x_{i},y_{i}\right)$ is taken from distribution D with an i.i.d. condition. We define the training set loss as $L_{S}\left(w\right):=\frac{1}{n}\sum_{i=1}^{n}l\left(w,x_{i},y_{i}\right)$ and the population loss as $L_{D}\left(w\right):=E_{(x,y)\;D}\left[l\left(w,x_{i},y_{i}\right)\right]$. By monitoring only S, our goal during model training is to select model parameters that have low population loss $L_{D}\left(w\right)$. This implies that instead of seeking FR-UNet parameters that have low training loss values, we use SAM to seek parameters for which the entire neighborhoods have uniformly low training loss values (similarly, neighborhoods with low loss and low curvature). This is inspired by the relationship between generalization and the loss landscape’s sharpness. The following shows the theorem of how SAM is integrated into the FR-UNet model. Figure 1 shows the block diagram of how SAM is integrated into the RF-UNet model.

After initialization of the model, its parameter values are selected by solving the following sharpness-aware minimization (SAM) problem:

(1) $$\min_{w}L_{S}^{\mathrm{SAM}}\left(w\right)+{\lambda ∥w∥}_{2}^{2}\;\mathrm{where}\;L_{S}^{\mathrm{SAM}}(w:=\max_{{∥\epsilon )∥}_{p}\leq \rho }L_{S}\left(w+\epsilon \right),$$

where ρ≥0 and is a hyperparameter representing the neighborhood radius where SAM seeks its loss, and p∈[1,∞].

SAM is integrated into RF-UNet by applying its base optimizer (Adam) directly to the SAM objective, and $L_{S}^{\mathrm{SAM}}\left(w\right)$ has to be minimized to achieve this. Minimization of $L_{S}^{\mathrm{SAM}}\left(w\right)$ can be done by deriving an efficient and effective approximation to $\nabla_{w}L_{S}^{\mathrm{SAM}}\left(w\right)$ by differentiating through the inner maximization. Following this path, the inner maximization problem is firstly approximated via Taylor expansion of $L_{S}\left(w+\epsilon \right)$ with respect to ϵ around 0, obtaining

(2) $$\epsilon *\left(w\right):=\underset{{∥\epsilon )∥}_{p}\leq \rho }{\arg\max}L_{S}\left(w+\epsilon \right)\approx \underset{{∥\epsilon )∥}_{p}\leq \rho }{\arg\max}L_{S}\left(w\right)+\epsilon^{T}\nabla_{w}L_{S}\left(w\right)=\underset{{∥\epsilon )∥}_{p}\leq \rho }{\arg\max}\epsilon^{T}\nabla_{w}L_{S}\left(w\right).$$

In turn, the value $\overset{\wedge }{\epsilon }\left(w\right)$ that solves this approximation is given by the solution to a classical dual norm problem (${\left|.\right|}^{q-1}$ denotes the elementwise absolute value and power):

(3) $$\overset{\wedge }{\epsilon }\left(w\right)=\rho \mathrm{sign}\left(\nabla_{w}L_{S}\left(w\right)\right){\left|\nabla_{w}L_{S}\left(w\right)\right|}^{q-1}/{\left(∥\nabla_{w}L_{S}{\left(w\right)∥}_{q}^{q}\right)}^{1/p}$$

where 1/p + 1/q = 1. Substituting back into Equation (3) and differentiating, we have

$$\begin{array}{cc} \nabla_{w}L_{S}^{\mathrm{SAM}}\left(w\right) & \approx \nabla_{w}L_{S}\left(\left(w\right)+\overset{\wedge }{\epsilon }\left(w\right)\right)=\frac{d(w+\overset{\wedge }{\epsilon }\left(w\right))}{dw}\nabla_{w}L_{S}{\left(w\right)|}_{w+\overset{\wedge }{\epsilon }\left(w\right)}\\ & =\nabla_{w}L_{S}\left(w\right){{|}_{w+\overset{\wedge }{\epsilon }\left(w\right)}+\frac{d\overset{\wedge }{\epsilon }\left(w\right)}{dw}\nabla_{w}L_{S}\left(w\right)|}_{w+\overset{\wedge }{\epsilon }\left(w\right)}. \end{array}$$

To speed up the approximation of $\nabla_{w}L_{S}^{\mathrm{SAM}}\left(w\right)$, second-order terms are dropped, providing our final gradient approximation:

(4) $$\nabla_{w}L_{S}^{SAM}\left(w\right)\approx \nabla_{w}L_{S}\left(w+\overset{\wedge }{\epsilon }\left(w\right)\right)$$

**Figure 2:**
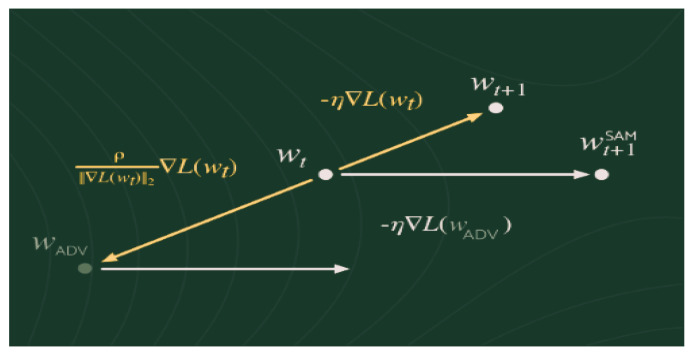
Single SAM parameter update.

The final SAM algorithm is obtained by applying a standard numerical optimizer—in our case, Adam—to the SAM objective $L_{S}^{\mathrm{SAM}}\left(w\right)$, and we use Equation (4) to calculate the required objective function gradients. Algorithm 1 following is the pseudo-code for the SAM algorithm integrated into the RF-UNet Adam optimizer, and Figure 2 schematically illustrates a single SAM parameter update.   
**Algorithm 1:** SAM-Enhanced RF-UNet Training Process
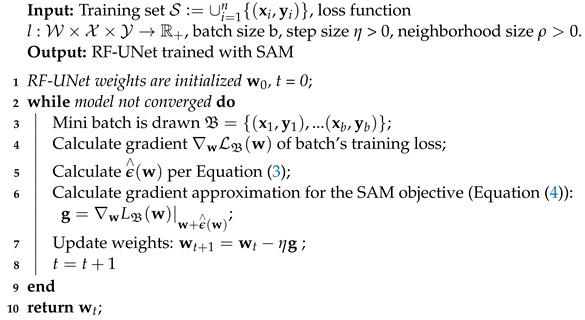


## 3. Results

In this section, we present the results of our experiments on retinal vessel segmentation using sharpness-aware minimization (SAM). We compare the performance of our RF-UNet trained with SAM to RF-UNet trained without SAM. Our comparison focuses on key metrics such as accuracy (Acc), sensitivity (Sen), Specificity (Spe), F1 score (F1), intersection over union (IOU), and the area under the receiver operating characteristic curve (AUC).

### 3.1. Dataset

**Figure 3:**
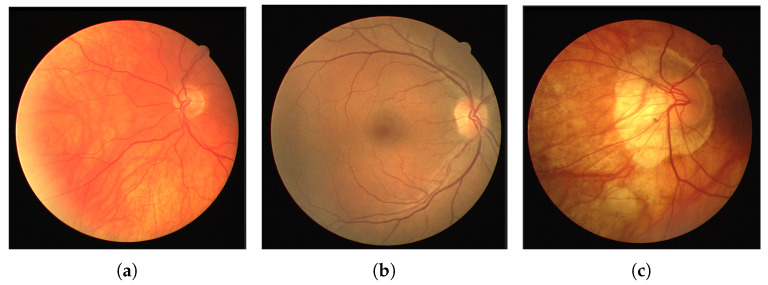
A sample of images from the DRIVE dataset. (**a**) an image having the maximum brightness. (**b**,**c**) images with the low brightness.

The images for the DRIVE dataset [42] were taken from a diabetic retinopathy screening program in the Netherlands. The screening population contained 400 diabetic volunteers aged 25 to 90 years. From 40 randomly selected images, 7 show signs of mild early diabetic retinopathy and 33 do not show any signs of diabetic retinopathy. The set of 40 images is divided into a training set and a testing set, both containing 20 images. There is only one manual segmentation of the blood vessel for training images. Two manual segments are available for test cases: one is used as a reference, and the other can be used to compare computer-generated segments with those of an independent observer. Additionally, mask images that indicate the region of interest are available for each retinal image. A sample of DRIVE dataset images is shown in Figure 3 below.

### 3.2. Metrics and Evaluation

We used a range of metrics in this study to assess our model’s performance. Some of these metrics are accuracy (Acc), sensitivity (Sen), specificity (Spe), F1 score, and area under the receiver operating characteristic curve (AUC); we made sure to capture both the advantages and disadvantages of our model using various performance dimensions. We go into the definition of each metric below:

(5) $$Accuracy=\frac{TP+TN}{TP+TN+FP+FN}$$

(6) $$Sensitivity=\frac{TP}{TP+FN}$$

(7) $$Specificity=\frac{TN}{TN+FP}$$

(8) $$F1=2\times \frac{Precision\times Recall}{Precision+Recall}$$

The symbols TP, TN, FP, and FN stand for the number of true positives, the number of true negatives, the number of false positives, and the number of false negatives, respectively.

### 3.3. Experimental Setup

We implemented our proposed method with PyTorch and ran experiments on a G3 3590 Dell computer with a GeForce GTX 1650 GPU. We conducted two experiments. For the first one, we trained FR-UNet with Adam as its base optimizer, and for the second experiment, we trained FR-UNet and integrated it with SAM as its optimizer. During the training for the second experiment, the standard numeric optimizer used in FR-UNet (Adam) was added to the SAM objective to calculate the required objective function gradient. SAM deals with minimizing the loss value and loss sharpness. SAM has a neighborhood radius (ρ) as its hyperparameter; for our case, we used ρ=0.05 since this leads to more stable convergence. Both experiments were trained for 30 epochs with a learning rate of 1 × 10${\;\;}^{-6}$ and a weight decay of 1 × 10${\;\;}^{-5}$. The RF-UNet code can be found at https://github.com/lseventeen/FR-UNet (accessed on 4 June 2024), and we used the code from https://github.com/davda54/sam (accessed on 4 June 2024) to learn how to integrate SAM into a model.

### 3.4. Experimental Results with SAM

As stated earlier, we used RF-UNet as the base model and integrated it with SAM. In this section, we show the results that we obtained after training the model for 30 epochs; we observed the following average performance metrics, which are also shown in Table 1. The average loss after 30 epochs was 0.094225. This relatively low loss indicates that the model effectively minimized the error during training, suggesting a good fit for the training data with the accuracy of 0.96225. This high accuracy demonstrates that the model correctly classified the majority of pixels in the retinal images, indicating strong overall performance. The AUC of 0.97987 reflects excellent model performance in distinguishing between retinal vessels and non-vessel regions. The F1 score of 0.79926 indicates a good balance between precision and recall in our segmentation task. The sensitivity (recall) of the model was 0.76249. This value shows that the model is able to identify 76.25% of the actual vessel pixels. While this is a reasonably high sensitivity, there is room for improvement to ensure more true vessel pixels are correctly identified. The specificity was 0.98437, indicating that the model correctly identified 98.44% of the non-vessel pixels. This is crucial in medical imaging because it minimizes false positives, which could lead to incorrect diagnoses or unnecessary treatments.

### 3.5. Comparison to the Model Trained without SAM

**Figure 4:**
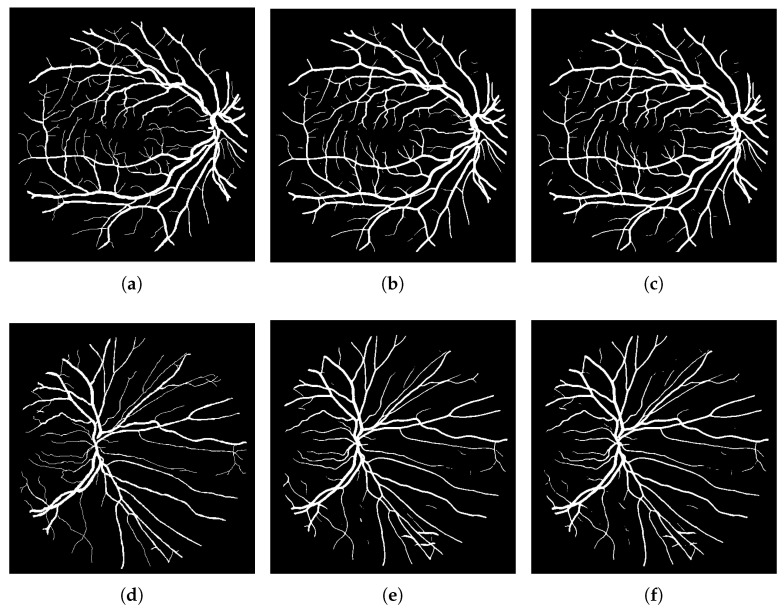
This figure shows visual comparison between the ground truths, output of RF-UNet without SAM and output of FR-UNet with SAM: (**a**,**d**) ground truth images from DRIVE dataset, (**b**,**e**) output images of RF-UNet without SAM, and (**c**,**f**) output images of RF-UNet with SAM.

In order to assess the efficacy of sharpness-aware minimization (SAM) for retinal vessel segmentation, we conducted a comparative analysis between the RF-UNet trained with SAM and the RF-UNet trained without SAM over a 30-epoch period. Table 2 summarizes the comparison of the key performance metrics: precision (Pre), sensitivity (Sen), specificity (Spe), loss, accuracy (Acc), area under the curve (AUC), F1 score (F1), and precision (Pre). Figure 4 shows a comparison between the retinal vessel segmentation images obtained after testing with RF-UNet trained with SAM, with RF-UNet trained without SAM, and the ground truths of the DRIVE test dataset.

### 3.6. Impact of Sharpness-Aware Minimization on Generalization

We compared the validation results of RF-UNet trained with sharpness-aware minimization (SAM) to the results of RF-UNet trained without SAM to evaluate the impact of SAM on the generalization achievement of the RF-UNet model for retinal vessel segmentation. Table 3 displays the outcomes of the key performance metrics.

The validation results for retinal vessel segmentation presented in Table 3 make it evident that sharpness-aware minimization greatly enhances the RF-UNet model’s generalization performance. The model learns more robust features that effectively generalize to unknown data with the assistance of SAM, as evidenced by the improvements in loss, accuracy, AUC, F1 score, sensitivity, and specificity.

The substantial 80% reduction in loss proves that the SAM-trained model is less prone to overfitting and better captures the underlying patterns in the data, leading to improved performance on the validation set. The 3% increase in accuracy suggests that SAM enhances the model’s ability to generalize well to new, unseen data. A higher AUC indicates that the SAM-trained model has a better ability to discriminate between vessel and non-vessel pixels across different threshold settings, reflecting superior generalization performance. The 36.43% improvement in F1 score demonstrates that the SAM-trained model maintains a better balance between identifying true positive and true negative pixels, which is crucial for reliable segmentation performance. The increase in sensitivity indicates that the SAM-trained model is significantly better at detecting actual vessel pixels to reduce the rate of false negatives, which is critical for accurate medical diagnoses. Lastly, the slight improvement in specificity shows that the SAM-trained model maintains a high ability to correctly identify non-vessel pixels to reduce false positives, which is essential for precise segmentation.

The improved performance of the SAM-trained model in all important metrics highlights how well SAM improves model generalization, making it a useful method for the progression of retinal vessel segmentation.

## 4. Discussion

In this study, we implemented sharpness-aware minimization (SAM) to improve the RF-UNet model’s generalization performance for retinal vessel segmentation. Our findings show that adding SAM to the training procedure significantly improves key performance metrics. More specifically, when compared to the model trained without SAM, the model trained with SAM performed better on both the training and validation datasets. This section explores the wider implications of these findings, places them in the context of our working hypotheses and earlier research, and makes recommendations for future research.

Prior research on retinal vessel segmentation has mostly concentrated on different deep learning architectures and optimization strategies to enhance the robustness and accuracy of the model. For example, because of the strong feature extraction and localization capabilities of standard U-Net and its variants, they have been used widely. But these models frequently suffer from overfitting, especially when they are trained on the small datasets that are frequently used in medical imaging.

Our results are consistent with recent studies that show the advantages of advanced optimization techniques such as SAM for reducing overfitting and improving generalization. Our study’s notable reduction in training and validation loss (by roughly 79.39% and 80.00%, respectively) is in line with gains documented in related studies using SAM in other domains. The improvements in accuracy (training accuracy rising by 6.71% and validation accuracy by 3.00%) provide more evidence that SAM is a useful tool for building robust image segmentation models.

**Figure 5:**
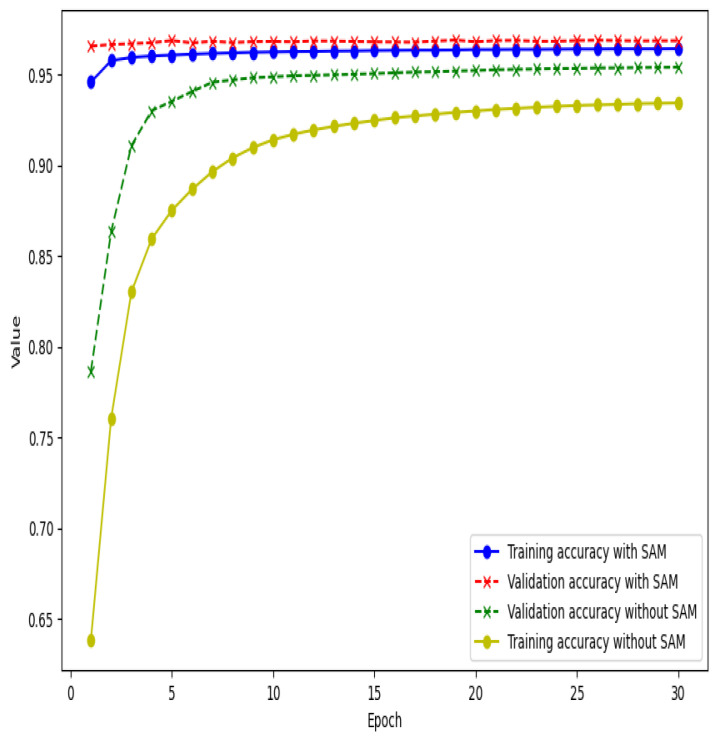
Validation and training accuracy through 30 epochs of RF-UNet trained with SAM and without SAM.

Our working hypothesis was that by facilitating a flatter, more generalizable minima in the loss landscape, adding SAM would enhance the RF-UNet model’s capacity for generalization. The substantial improvements observed in all key metrics, such as accuracy, AUC, F1 score, sensitivity, and specificity, provide evidence in favor of this hypothesis. The improved training and validation accuracies show that the SAM-trained model reduces overfitting by learning the training data more successfully and applying that knowledge to new data. Figure 5 shows the comparison of the training and validation data results for RF-UNet trained using SAM and without SAM. The rest of the graphs that show our comparison of the training and validation results for other metrics over 30 epochs are found in Appendix A. As you can see from Figure 5 above, the model trained with SAM outperformed the other model without SAM in terms of both better validation and training, hence, providing more proof of SAM improving generalization.

Furthermore, the average training accuracy increased from 0.90169 to 0.96225, indicating that SAM aids with the model’s more effective capture of the underlying patterns in the data. This improvement can probably be attributed to SAM’s capacity to identify flatter minima, which are typically linked to superior generalization. Also, the average validation accuracy increased from 0.93999 to 0.96821, indicating that the model can now more broadly generalize to new data. This is especially crucial for medical imaging, where the model needs to function consistently for a variety of previously unobserved images.

The field of medical image analysis will be significantly impacted by these findings. SAM enhancement improves generalization: it not only improves retinal vascular segmentation model performance but may also find use in other medical imaging tasks like organ segmentation and tumor detection. For clinical applications, where models need to be resilient to varying imaging conditions and patient demographics, good generalization is essential.

In addition, notable improvements were made with SAM in terms of specificity (from 0.97620 to 0.98265) and sensitivity (from 0.53305 to 0.79861). High sensitivity minimizes the possibility of missed detection, ensuring the accurate identification of true vessel pixels—a crucial component for precise medical diagnosis. High specificity also lessens false positives, which lowers the possibility of making the wrong treatment choices.

To conclude, we found that although sharpness-aware minimization (SAM) greatly improves the performance metrics of the model, there are not many noticeable changes in the visual output. In particular, quantitative gains in metrics like accuracy, specificity, and sensitivity show how well SAM refines RF-UNet’s performance. However, as you can see from Figure 4, it appears that there were only minor improvements in boundary delineation and edge sharpness between the visual segmentation results obtained before and after applying SAM. This suggests that the impact of SAM is more evident in the numerical performance indicators than in the observable visual improvements. As such, SAM helps create a more stable and dependable model, but its benefits might not be immediately apparent from a visual inspection. Our future research could explore the following directions:

- Testing our SAM-enhanced model on other diverse retinal image datasets such as CHUAC, STARE, and DCA1.
- Combining SAM with other cutting-edge methods like transfer learning and data augmentation to optimize model performance. Further research into the synergistic effects of these techniques may produce even more improved generalization.
- Extending the application of SAM to other medical imaging tasks such as MRI and CT scan analysis could show off its adaptability to and efficiency in a variety of contexts.
- Conducting a systematic investigation on how various hyperparameters may affect SAM’s performance in order to better optimize the training procedure for some tasks.
- Evaluating the SAM-trained model in real-world clinical settings.

## 5. Conclusions

In this work, we examined the effect of sharpness-aware minimization (SAM) on the retinal vessel segmentation generalization performance of the RF-UNet model. According to our experimental findings, the model performs much better across various key metrics such as accuracy, AUC, F1 score, sensitivity, specificity, and loss when SAM is included.

In particular, the SAM-trained RF-UNet model (training loss of 0.094225 and validation loss of 0.08053), perform much better than the non-SAM model (training loss of 0.45709 and validation loss of 0.40266), suggesting better convergence and less overfitting using SAM. Additionally, the model’s improved capacity to generalize to new data is demonstrated by the increase in validation accuracy (from 0.93999 to 0.96821) and training accuracy (from 0.90169 to 0.96225). The noteworthy enhancements in AUC, F1 score, sensitivity, and specificity accentuate the efficacy of SAM in generating dependable and resilient segmentation models.

Our results corroborate our hypothesis that SAM facilitates the learning of flatter minima, which leads to better generalization, and they are consistent with recent research showing the advantages of sophisticated optimization techniques in deep learning. The enhanced performance measures imply that SAM can reduce overfitting and improve the model’s suitability for use in actual clinical situations.

Our subsequent studies ought to examine the utilization of SAM on more extensive and varied datasets, examine the amalgamation of SAM with additional optimization and augmentation methodologies, and authenticate the model’s efficacy in authentic clinical settings.

To sum up, sharpness-aware minimization (SAM) offers a promising way to improve the accuracy and robustness of medical image analysis models by considerably enhancing the generalization capability of the RF-UNet model for retinal vessel segmentation. This work sets the stage for future developments in the area, which will eventually lead to the development of more dependable and efficient diagnostic instruments for the medical field.

## Figures and Tables

**Table 1:** Average performance metrics after 30 epochs.

Metric	Value
Loss	0.094225
Acc	0.96225
Auc	0.97987
F1	0.79926
Sen	0.76249
Spe	0.98437

**Table 2:** Comparison of RF-UNet trained with SAM and RF-UNet trained without SAM.

Metric	with SAM	without SAM	Improvement
Loss	0.09487	0.45709	+79.39%
Acc	0.96225	0.90169	+6.71%
Auc	0.97987	0.85756	+14.28%
F1	0.79926	0.55216	+44.82%
Sen	0.76249	0.56381	+35.26%
Spe	0.98437	0.93909	+4.82%

**Table 3:** Validation performance comparison of RF-UNet with and without SAM.

Metric	with SAM	without SAM	Improvement
Loss	0.08053	0.40266	+80.00%
Acc	0.96821	0.93999	+3.00%
Auc	0.98388	0.89083	+10.38%
F1	0.79322	0.58144	+36.43%
Sen	0.79861	0.53305	+49.84%
Spe	0.98265	0.97620	+0.66%

**Figure A1:**
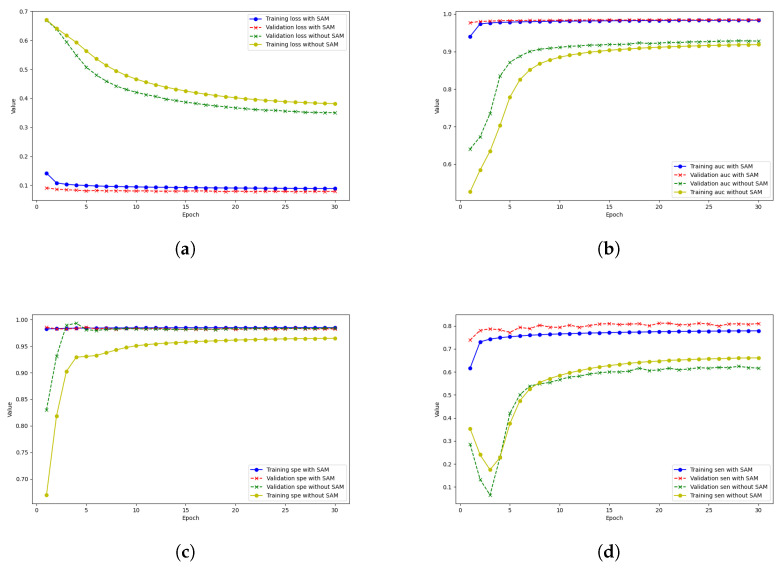
(**a**) Validation loss and training loss of both SAM-trained and non-SAM-trained, (**b**) validation Auc and training Auc of both SAM-trained and non-SAM-trained, (**c**) validation Spe and training Spe of both SAM-trained and non-SAM-trained, and (**d**) validation Sen and training Sen of both SAM-trained and non-SAM-trained.

## Data Availability

Data is contained within the article.

## Abbreviations

The following abbreviations are used in this manuscript:

SAM	sharpness-aware minimization
Acc	accuracy
F1	F1 score
Sen	sensitivity
Spe	specificity

## Appendix A

Below we show the comparison of the results obtained by the SAM-trained RF-UNet model with the non-SAM RF-UNet over a period of 30 epochs.
